# Regional Brain Aging Disparity Index: Region-Specific Brain Aging State Index for Neurodegenerative Diseases and Chronic Disease Specificity

**DOI:** 10.3390/bioengineering12060607

**Published:** 2025-06-03

**Authors:** Yutong Wu, Shen Sun, Chen Zhang, Xiangge Ma, Xinyu Zhu, Yanxue Li, Lan Lin, Zhenrong Fu

**Affiliations:** 1 Department of Biomedical Engineering, College of Chemistry and Life Sciences, Beijing University of Technology, Beijing 100124, China; wyt191026@emails.bjut.edu.cn (Y.W.); sunshen@bjut.edu.cn (S.S.); zc202265180@emails.bjut.edu.cn (C.Z.); maxiangge@emails.bjut.edu.cn (X.M.); zhuxyu@emails.bjut.edu.cn (X.Z.); liyanxue@emails.bjut.edu.cn (Y.L.); 2 Intelligent Physiological Measurement and Clinical Translation, Beijing International Base for Scientific and Technological Cooperation, Beijing University of Technology, Beijing 100124, China; 3 School of Psychology, Central China Normal University, Wuhan 430079, China; 4 Key Laboratory of Human Development and Mental Health of Hubei Province, Wuhan 430079, China; 5 Key Laboratory of Adolescent Cyberpsychology and Behavior, Ministry of Education, Wuhan 430079, China

**Keywords:** model interpretability, SHAP, brain age prediction, deep learning, abnormal brain aging

## Abstract

This study proposes a novel brain-region-level aging assessment paradigm based on Shapley value interpretation, aiming to overcome the interpretability limitations of traditional brain age prediction models. Although deep-learning-based brain age prediction models using neuroimaging data have become crucial tools for evaluating abnormal brain aging, their unidimensional brain age–chronological age discrepancy metric fails to characterize the regional heterogeneity of brain aging. Meanwhile, despite Shapley additive explanations having demonstrated potential for revealing regional heterogeneity, their application in complex deep learning algorithms has been hindered by prohibitive computational complexity. To address this, we innovatively developed a computational framework featuring efficient Shapley value approximation through a novel multi-stage computational strategy that significantly reduces complexity, thereby enabling an interpretable analysis of deep learning models. By establishing a reference system based on standard Shapley values from healthy populations, we constructed an anatomically specific Regional Brain Aging Deviation Index (RBADI) that maintains age-related validity. Experimental validation using UK Biobank data demonstrated that our framework successfully identified the thalamus (THA) and hippocampus (HIP) as core contributors to brain age prediction model decisions, highlighting their close associations with physiological aging. Notably, it revealed significant correlations between the insula (INS) and alcohol consumption, as well as between the inferior frontal gyrus opercular part (IFGoperc) and smoking history. Crucially, the RBADI exhibited superior performance in the tri-class classification of prodromal neurodegenerative diseases (HCs vs. MCI vs. AD: AUC = 0.92; HCs vs. pPD vs. PD: AUC = 0.86). This framework not only enables the practical implementation of Shapley additive explanations in brain age prediction deep learning models but also establishes anatomically interpretable biomarkers. These advancements provide a novel spatial analytical dimension for investigating brain aging mechanisms and demonstrate significant clinical translational value for early neurodegenerative disease screening, ultimately offering a new methodological tool for deciphering the neural mechanisms of aging.

## 1. Introduction

Aging, a multifaceted biological process, is characterized by a gradual decline in physiological functions over time. While chronological age (CA) serves as a straightforward metric for quantifying the passage of time, it fails to account for significant individual variability in the rate of aging [1]. This discrepancy has led to the development of the concept of biological age (BA), which provides a more nuanced measure by reflecting an individual’s physiological state relative to their chronological age [2,3]. Biological age is associated with a range of influencing factors, including genetic predisposition, lifestyle choices, and environmental exposures, offering a more individualized assessment of the aging process [4,5]. The divergence between CA and BA is often categorized as accelerated or delayed aging, where BA exceeds or falls short of CA, respectively. This dynamic approach has proven particularly valuable when applied to specific biological systems or organs, with the brain emerging as a critical area of focus. Brain aging spans the entire lifespan and is characterized by intricate, heterogeneous patterns of structural and functional changes. These changes, while influenced by natural aging processes, are also shaped by pathological conditions, such as neurodegenerative diseases. Understanding these variations is essential for differentiating between normal and abnormal brain aging trajectories. Key structural alterations in the brain include reductions in cortical thickness, surface area, and gray matter (GM) volume. These changes do not occur uniformly but vary across brain regions, reflecting the complexity of aging at the morphological level. Additionally, pathological processes such as Alzheimer’s disease (AD) or Parkinson’s disease (PD) exacerbate these structural declines. For instance, regions like the hippocampus and amygdala, which are critical for memory and emotional processing, show pronounced atrophy in AD. Such changes underscore the need for precise biomarkers that can capture deviations from typical aging patterns [6,7]. Brain BA has emerged as a valuable biomarker for assessing these deviations [8]. Advances in machine learning have facilitated the prediction of brain BA using neuroimaging data, with a particular focus on techniques such as T1-weighted MRI. The discrepancy between predicted brain BA and CA, termed the brain age gap (BAG), provides a straightforward assessment of abnormal brain aging. A positive BAG—indicating that brain BA exceeds CA—has been associated with various neurodegenerative conditions, including mild cognitive impairment (MCI) [9], Alzheimer’s disease (AD) [10], and Parkinson’s disease (PD) [11]. This metric offers insights into early disease detection and progression, contributing to both clinical decision making and the broader understanding of brain aging mechanisms. The application of BAG extends beyond diagnosis. It has proven instrumental in tracking longitudinal changes in brain health, differentiating between normal aging trajectories and those affected by neurodegenerative diseases. For example, individuals with MCI often exhibit a positive BAG, and monitoring its progression can help predict the likelihood of conversion to AD. Similarly, in PD, brain BA assessments can shed light on disease-related morphological alterations, aiding in both early detection and personalized treatment planning.

While BAG has emerged as a promising biomarker for aging and neurodegenerative diseases, it has limitations in providing detailed insights into the underlying neurobiological mechanisms. A primary limitation is its lack of spatial specificity. BAG is typically calculated as a single scalar value, representing the overall brain age. This approach fails to capture regional variations in brain aging, which can be crucial for understanding disease-specific patterns of neurodegeneration. For instance, while thalamic volume progressively declines after adulthood in healthy individuals, hippocampal volume follows a non-linear trajectory, increasing initially before decreasing later in life [5]. Furthermore, distinct neurodegenerative diseases exhibit characteristic patterns of regional atrophy; for example, the medial temporal lobe is severely affected in early AD [12], whereas PD studies are associated with accelerated atrophy in the striatum [13]. These regional variations underscore the inability of BAG, which focuses on global metrics, to capture disease-specific changes. Efforts to develop regionally specific brain age models, such as those proposed by Nguyen et al. and Kaufmann et al., have aimed to address these limitations [14,15]. However, these models often suffer from limited granularity in neuroanatomical detail, which can hinder their accuracy when regions with low age-related relevance are included. Recent advances in model interpretation methods offer new opportunities for understanding regional differences in brain aging. These methods quantify the contribution of individual neuroimaging features to the predictive model, enabling the identification of disease-relevant regions [16,17]. Among the widely used approaches, Class Activation Mapping (CAM) [18] and saliency map techniques [19,20] provide visual explanations by highlighting important features from specific layers of deep learning models. Despite their utility, these methods are constrained by their dependence on specific network architectures, limiting their broader applicability [21,22]. In contrast, Shapley additive explanations (SHAP) offer a model-agnostic framework based on game theory to quantify the contribution of individual features [23,24,25]. SHAP has been widely adopted in machine learning applications for brain age prediction due to its ability to generate robust and interpretable feature importance scores [26]. However, applying SHAP to deep learning models presents significant challenges due to its computational complexity, which scales exponentially with the number of features ($2^{n}$, where *n* is the number of features). This limitation renders regional-level interpretations for deep learning models nearly infeasible, leaving a critical gap in the application of SHAP for understanding brain aging at a finer anatomical scale. Therefore, the present study aims to develop an efficient SHAP-based method for brain-region-specific aging assessment and to investigate its potential in detecting early-stage manifestations of neurodegenerative disorders.

**Figure 1:**
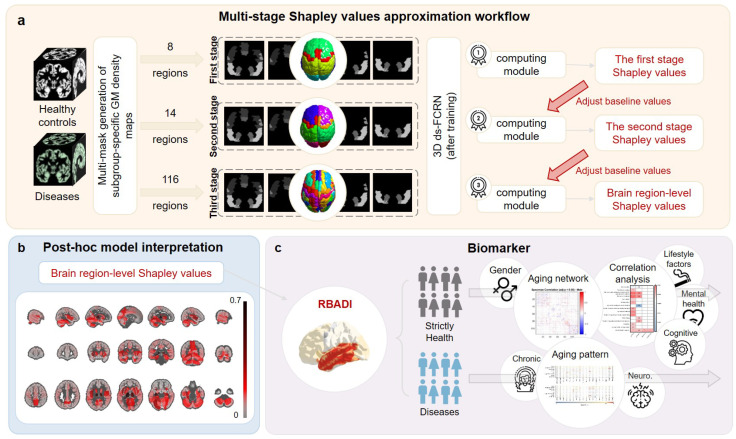
The overall workflow of this study. Part (**a**) illustrates the multi-stage Shapley values approximation workflow; part (**b**) demonstrates the method for calculating brain region contributions through Shapley value attribution; part (**c**) presents a series of experiments conducted using the developed RBADI. For healthy subjects, the focus is on identifying lifestyle factors that influence brain aging regions and exploring gender-related aging networks, while for diseased subjects (MCI, AD, pPD, PD, and chronic conditions), the primary aim is to identify aging patterns that differ from those of healthy subjects.

In this study, we made two key contributions, as outlined in Figure 1.

First, we propose a novel multi-stage Shapley values approximation approach that significantly reduces computational complexity. Compared with the standard Shapley value computation method, our approach based on the automated anatomical labeling (AAL) 116 template achieves a computational complexity reduction of 30 orders of magnitude, thereby enabling feature contribution analysis at the brain region level in deep learning models.

Second, leveraging the insights derived from multi-stage Shapley values approximation, we developed the regional brain aging disparity index (RBADI) to quantify the regional brain aging status of an individual relative to their peers. This metric facilitates the identification of environmental and lifestyle factors contributing to abnormal brain aging in healthy populations, thereby informing personalized health recommendations. Furthermore, it demonstrates an excellent performance in the early detection of neurodegenerative diseases (AD and PD), exhibiting promising clinical application prospects for timely intervention and disease management.

## 2. Materials and Methods

### 2.1. Database

This study utilized data from three publicly available datasets: the UK Biobank (UKB), the Alzheimer’s Disease Neuroimaging Initiative (ADNI), and the Parkinson’s Progression Markers Initiative (PPMI). Ethical approval from the Human Research Ethics Committee at each location was obtained prior to the start of this study. Specifically, T1 MRI data from 22,149 participants in the UKB database were included. These scans were acquired using 3D MPRAGE, with an in-plane acceleration of iPAT = 2 and pre-scan normalization. The image size is 208×256×256 voxels, with a voxel resolution of 1×1×1 ${\mathrm{mm}}^{3}$. Participants diagnosed with psychiatric disorders, as identified by ICD-10 codes (the exclusion criteria are presented in Supplementary Table S1), and those with poor quality or with obvious artifacts were excluded. Ultimately, 16,377 healthy participants (including chronic conditions participants) aged 45 to 82 were retained for this study. For the ADNI database, T1 MRI data from 582 healthy controls (HCs), 432 MCI participants, and 252 AD participants were included. For the PPMI database, T1 MRI data from 36 HCs, 426 prodromal PD (pPD) participants, and 264 PD participants were included. The UKB data were used to train, validate, and test the deep learning model. The ADNI and PPMI data were employed to analyze differences between neurodegenerative conditions—MCI, AD, pPD, and PD—and HCs. Demographic information for those databases is shown in Table 1.

### 2.2. Image Preprocessing

The T1 MRI data from all three datasets (UKB, ADNI, and PPMI) underwent a standardized processing pipeline using the CAT12 (a Computational Anatomy Toolbox for SPM, https://neuro-jena.github.io/cat/ (accessed on 20 August 2023)) tool, seamlessly integrated within SPM12 (Statistical Parametric Mapping, https://www.fil.ion.ucl.ac.uk/spm/software/ (accessed on 20 August 2023)). The pipeline included brain extraction, tissue segmentation, and spatial normalization. The brain extraction removed non-brain tissue such as the skull and scalp, isolating the brain for subsequent analysis. Tissue segmentation delineated the brain into GM, white matter, and cerebrospinal fluid. Spatial normalization aligned individual brains to a common template space. CAT12 computes the GM density at the voxel level by aligning the segmented GM image to a standard template and determining the proportion of GM in each voxel. Quality control was rigorously carried out throughout the process, using both the overall weighted image quality (IQR) score provided by CAT12 and visual inspection. Images with an IQR score of ‘C-’ or lower were strictly excluded. Those scoring ‘C’ or higher underwent a thorough visual inspection to exclude any images with visible artifacts or quality issues, ensuring the accuracy of segmentation. This comprehensive preprocessing led to the generation of GM density maps, with a voxel resolution of 1×1×1 ${\mathrm{mm}}^{3}$ and dimensions of 169×205×169 voxels. Given the well-established association between GM density and brain aging, and supported by previous studies demonstrating the superior performance of models trained on GM density maps for brain age prediction [2,27,28,29], GM density maps were selected as the input for our model. To reduce computational demands during model training, a trilinear interpolation method was employed to down-sample the GM density maps to a resolution of 84×102×84 voxels, with a voxel size of 2×2×2 ${\mathrm{mm}}^{3}$. The data from the UKB database were then divided into training, validation, and test sets in a ratio of 6:2:2, containing 9826 (mean age, 64.31±7.55, 4698 men), 3275 (mean age, 64.30±7.49, 1552 men), and 3276 (mean age, 64.15±7.45, 1561 men) healthy subjects, respectively. The data from the ADNI and PPMI databases were not included in the model training and validation processes. The undivided datasets from these two databases were specifically reserved as an external test set to rigorously evaluate the model’s generalization capability.

### 2.3. Brain Age Prediction Model

**Figure 2:**
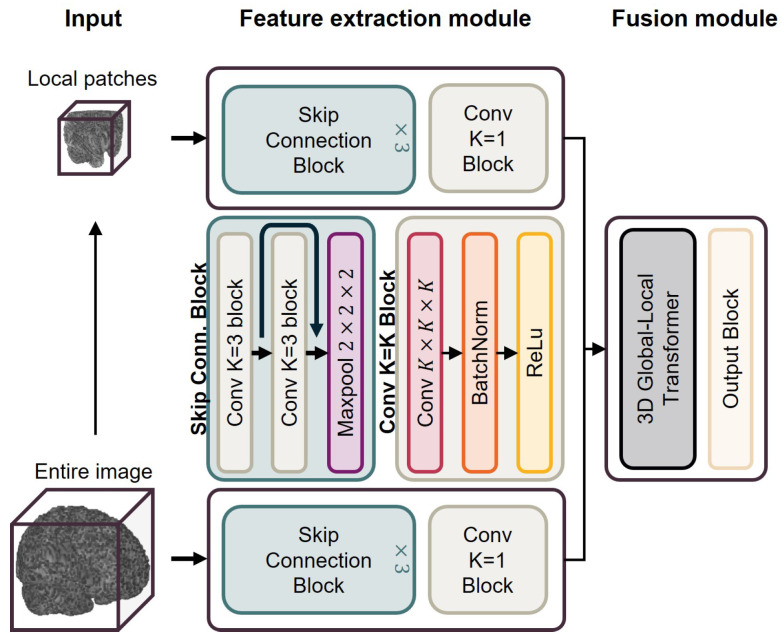
Model architecture. The entire GM density map is divided into local GM density patches using a sliding window approach. Patches and entire maps undergo feature extraction through independent feature extraction modules, respectively. The extracted features are then fused by a fusion module and mapped to brain age. The detailed architecture of the model is presented in previous research [30].

A 3D dual-stream fully convolutional residual network (3D ds-FCRN) is employed to model GM density maps for predicting brain age. This network is characterized by an efficient dual-stream feature extraction mechanism, which extracts features from the whole image and local patches [30]. The 3D global–local Transformer module integrates global and local features, demonstrating an excellent predictive performance and robustness. The specific architecture of the model is shown in Figure 2.

The model optimization was conducted using the Adam optimizer on the PyTorch platform (torch version 2.1.1+cu121), with an initial learning rate of 0.0001, while keeping the other parameters at their default settings. A learning rate scheduler was employed to reduce the learning rate by half every 5 epochs. Early stopping was implemented during training, with the maximum number of epochs set to 30. Training was terminated if the validation set performance did not improve for 5 consecutive epochs, and the model parameters were saved for further testing. The hyperparameters were set as follows: the patch size was 64, batch size was 6, and the number of global–local transformer blocks was 4. All training, validation, and testing were performed on a workstation equipped with an NVIDIA RTX 3090 GPU, an Intel Xeon W-2223 CPU, and 32 GB of RAM.

### 2.4. Multi-Stage Shapley Values Approximation Workflow

Shapley values, rooted in cooperative game theory, provide a robust framework for attributing the contributions of individual features to a model’s predictions. In brain age prediction tasks, Shapley values can be employed to characterize feature contributions by quantifying the marginal effects of distinct features (i.e., various brain regions) on predictive outcomes. Specifically, they reflect how individual neuroanatomical characteristics influence age estimation through their cooperative interactions within the predictive model. Previous studies have demonstrated that feature contribution vectors derived from Shapley values are capable of capturing biologically meaningful brain aging patterns, offering interpretable insights into neurodegenerative processes [25]. While effective, the computational cost of calculating exact Shapley values can be prohibitively high, particularly in high-dimensional datasets. To address this challenge, we propose a multi-stage approximation workflow designed to mitigate the computational complexity of SHAP. The workflow involves dividing the brain into anatomically meaningful regions and treating each region as an individual feature. By reducing the number of features, the computational cost of calculating Shapley values can be significantly reduced. This workflow is structured into three key stages. In the initial stage, the brain is segmented into eight anatomical regions using the AAL 116 template, which organizes the brain based on its anatomical structure. These regions include the temporal lobe (TL), frontal lobe (FL), prefrontal cortex (PFC), parietal lobe (PL), occipital lobe (OL), subcortical structures (SC), cerebellum (CB), and the cerebellar vermis (CV). The GM density maps corresponding to these regions are considered as individual features ($X_{st1}=\left(A_{TL},A_{FL},A_{PFC},A_{PL},A_{OL},A_{SC},A_{CB},A_{CV}\right),\;A_{\dot{j}}=\left\{f_{\dot{j}}\right\},\;\;\dot{j}\in Z⋂\left[1,8\right]$), providing a biologically informed basis for subsequent analysis and reducing the dimensionality of the input space. The Shapley values for the first stage are calculated using the following formula:

(1) $${\phi_{st1}}_{i\dot{j}}=\sum_{S\subseteq X_{st1}\dot{j}}\frac{\left|S\right|!\left(\left|X_{st1}\right|-\left|S\right|-1\right)!}{\left|X_{st1}\right|!}\left({v\left(S\cup \left\{\dot{j}\right\}\right)}_{i}-{v(S)}_{i}\right)$$

In this formula, ${\phi_{st1}}_{ij}$ represent the Shapley value for individual *i* in the $\dot{j}$-th brain region of the feature space $X_{st1}$; *S* denotes a subset of the feature space $X_{st1}$ excluding the $\dot{j}$-th brain region, with the number of features ranging from 0 to a maximum of 7; $\left|S\right|$ denotes the number of features in the feature space subset *S*, and similarly, $\left|X_{st1}\right|$ represents the number of features in $X_{st1}$, which is 8; ${v\left(S\cup \left\{\dot{j}\right\}\right)}_{i}$ represents the predicted outcome for individual *i* using the features in feature space subset *S* along with the $\dot{j}$-th brain region in the brain age prediction model, while ${v(S)}_{i}$ represents the predicted outcome for individual *i* using only the features in feature space subset *S*; and the subtraction of these two terms characterizes the marginal effect of the $\dot{j}$-th brain region within subset *S*. When the number of features in feature space subset *S* is 0, ${v(S)}_{i}$ is replaced with a baseline value ${E_{tr}}_{st1}$, which in this study is the mean CA of the subjects in the training set. The baseline value is consistent for all features in the feature space $X_{st1}$. In this framework, each brain region’s GM density map is treated as an independent feature. The contribution of each feature to brain age prediction is quantified through Equation (1) by computing its marginal effects across feature subset permutations of varying cardinalities, with subsequent aggregation of these effects. Therefore, for individual *i*, the Shapley values of all features in the feature space $X_{st1}$ should strictly satisfy the following formula:

(2) $$\sum_{\dot{j}=1}^{\left|X_{st1}\right|}{\phi_{st1}}_{i\dot{j}}={v(X_{st1})}_{i}-{E_{tr}}_{st1}$$

where ${v(X_{st1})}_{i}$ denotes the predicted outcome for individual *i* using all features in the feature space $X_{st1}$ in the brain age prediction model. For each subject, the summation of contributions from distinct brain regions must strictly equal the difference between the model-predicted age and the baseline value. In the second stage of the workflow, to further refine the analysis, the eight brain regions delineated in the first stage are further subdivided into fourteen regions based on hemispheric laterality (excluding the CV). This finer-grained division yields a feature space $X_{st2}$ ($X_{st2}=\left(A_{TL},A_{FL},A_{PFC},A_{PL},A_{OL},A_{SC},A_{CB}\right),\;A_{\ddot{j}}=\left\{f_{\ddot{j}l},f_{\ddot{j}r}\right\},\;\ddot{j}\in Z⋂\left[1,7\right]$). While this increased granularity provides a more detailed representation of the brain structure, it also significantly increases the computational complexity of calculating exact Shapley values. To address this challenge, an approximation method is employed in the second stage. The formula for calculating Shapley values in the second stage is as follows:

(3) $${\phi_{st2}}_{if_{\ddot{j}n}}=\sum_{S\subseteq A_{\ddot{j}}f_{\ddot{j}n}}\frac{\left|S\right|!\left(\left|A_{\ddot{j}}\right|-\left|S\right|-1\right)!}{\left|A_{\ddot{j}}\right|!}\left({v\left(S\cup \left\{f_{\ddot{j}n}\right\}\right)}_{i}-{v(S)}_{i}\right)$$

where $A_{\ddot{j}}$ represents the $\ddot{j}$-th feature set in the feature space $X_{st2}$. Thus, a total of 7 different feature sets need to be computed for Shapley values in the second stage. $f_{\ddot{j}n}$ denotes the *n*-th feature in the $\ddot{j}$-th feature set, corresponding to either the left ($f_{\ddot{j}l}$) or right ($f_{\ddot{j}r}$) hemisphere of the brain region represented by the $\ddot{j}$-th feature set. This approximate calculation method assumes that the Shapley values computed in the first stage accurately represent the true interactions among all features in the $X_{st1}$ feature space. In the second stage, each individual feature from the first stage is treated as a new feature set, and feature contributions are computed within each respective set. The baseline values are adjusted by utilizing the inter-set interactions obtained from the first stage to ensure strict equivalence in the computational process. The adjustment of the baseline value is given by the following formula:

(4) $${{E_{tr}}_{st2}}_{i\ddot{j}}={E_{tr}}_{st1}+\sum_{\dot{j}=1}^{X_{st1}CV,\;{\;A}_{\ddot{j}}}{\phi_{st1}}_{i\dot{j}}={v(X_{st1})}_{i}-{\phi_{st1}}_{i\ddot{j}}$$

where ${{E_{tr}}_{st2}}_{i\ddot{j}}$ represents the baseline value for individual *i* when considering the $\ddot{j}$-th feature set in the feature space $X_{st2}$. This approach approximates the influence of non-target features on the target feature by aggregating their Shapley values into the baseline value. Therefore, after calculating the Shapley values for each feature in all feature sets within the feature space $X_{st2}$, all Shapley values should strictly satisfy the following formula:

(5) $$\sum_{n=1}^{2}{\phi_{st2}}_{if_{\ddot{j}n}}={\phi_{st1}}_{i\ddot{j}}$$

Equation (5) characterizes that the summation within each individual feature set must strictly equal the Shapley values of the corresponding feature set computed in the first stage. For instance, the summation of the Shapley values for a subject’s left TL and right TL should exactly equal the Shapley value of the TL.

In the third stage, the brain regions are further refined into 116 distinct areas based on the AAL116 template. The GM density maps corresponding to these areas define the final feature space $X_{st3}$ ($X_{st3}=\left(A_{TL},A_{FL},A_{PFC},A_{PL},A_{OL},A_{SC},A_{CB},A_{CV}\right),\;A_{\dddot{j}}=\left\{f_{\dddot{j}1},f_{\dddot{j}2},\ldots ,f_{\dddot{j}n}\right\},\dddot{j}\in Z⋂\left[1,8\right]$). Similar to the computational strategy employed in the second stage, this step utilizes an approach that adjusts baseline values to streamline the calculation of marginal contributions between features, significantly reducing the computational complexity. Through the aforementioned three-step computational process, each participant was assigned a 116-dimensional feature contribution vector, where each dimension quantitatively corresponds to the Shapley value of a distinct brain region. Figure 1a provides a detailed overview of the computational workflow for this algorithm, illustrating the step-by-step process of feature decomposition and approximation. Supplementary Table S2 presents the division of brain regions across different stages, while Supplementary Table S3 provides a list of abbreviations for brain regions.

### 2.5. Model Interpretation and Regional Brain Aging Disparity Index (RBADI)

To quantify the contributions of individual brain regions to the age estimation process, we employed Shapley values derived from the third stage of our model. These Shapley values were used to construct the RBADI, a metric reflecting the degree of aging in each brain region relative to peers of the same chronological age. To investigate the impact of chronic diseases, we utilized the test subjects in the UKB dataset, identifying individuals with chronic diseases based on ICD-10 codes (the exclusion criteria are presented in Supplementary Table S4). This resulted in two distinct cohorts: a group of 592 subjects with chronic diseases (mean age 67.23±6.63, 334 men) and a control group of 2684 strictly healthy subjects (mean age 63.47±7.45, 1227 men). The strictly healthy subjects served as the reference group for establishing age-standard Shapley values and assessing the contributions of brain regions in healthy individuals. The method for calculating brain region contributions $C_{f_{\dddot{j}n}}$ is as follows:

(6) $$C_{f_{\dddot{j}n}}=\frac{1}{N}\sum_{i=1}^{N}\left|{\phi_{st3}}_{if_{\dddot{j}n}}\right|$$

where *N* represents the total number of subjects in the strictly healthy set. The calculation of age-standard Shapley values is performed using the following formula:

(7) $${\phi_{st3}}_{a}=\frac{1}{N_{\left[a-1,a+1\right]}}\sum_{i=1}^{N_{\left[a-1,a+1\right]}}{\phi_{st3}}_{i}$$

where ${\phi_{st3}}_{a}$ represents the age-standard Shapley value vectors at age *a*, and [a−1,a+1] denotes the age range limited to a−1 to a+1. For all subjects (HCs, chronic conditions, MCI, AD, pPD, and PD), their CA is rounded to the nearest integer. Then, the Shapley values at the third stage from each subject are subtracted by the corresponding age-standard Shapley values to obtain the RBADI.

### 2.6. Identification of Regional Brain Aging Patterns

A Spearman correlation analysis was used to investigate the factors influencing the RBADI of HCs. To control for potential confounding effects, gender and age were included as covariates and removed during the analysis. A comprehensive set of 146 lifestyle factors, encompassing early life experience (for example, birth weight, adoption, and childhood affective perception), sociodemographics (for example, education, job category, and commuting mode), lifestyle (for example, alcohol intake, smoking, diet, sleep, and exercise), psychosocial (for example, mental health and social support), general health (for example, menstrual cycle, hearing, and vision), and environmental exposures (for example, air and noise pollution), were considered (detailed in Supplementary Table S5). Data processing methods adhered to the procedures described by Tian et al. (heterogeneous aging across multiple organ systems and prediction of chronic disease and mortality). In total, 31 blood biochemical indicators were included in the correlation analysis, encompassing metabolic markers such as triglycerides and glucose, as well as sex hormone indicators such as estradiol and testosterone (detailed in Supplementary Table S6). The Free Androgen Index (FAI) was calculated using total testosterone and sex-hormone-binding globulin (SHBG) [31]. In total, 32 cognitive ability indicators were utilized to investigate the relationship between regional aging and cognition, including the results of ten tests representing different cognitive abilities, such as the Pairs Matching Test, Reaction Time Test, and Numeric Memory Test (detailed in Supplementary Table S7). Factors demonstrating a significant association with the RBADI were identified using a threshold of p<0.05 after a false discovery rate (FDR) correction.

To elucidate the influence of gender on brain aging networks, separate analyses were conducted for male and female subjects within the HC cohort. A Spearman correlation analysis was then applied to determine the correlation coefficients between brain regions within each gender group. Connections with a *p*-value of <0.05 after the FDR correction were considered significant, resulting in the construction of distinct brain aging networks of size 116×116 for males and females. All correlation analyses were performed using the MATLAB 2023b platform, with the FDR correction implemented using the Bioinformatics Toolbox within MATLAB.

To assess the impact of specific diseases on the RBADI, multivariate general linear models were used. Age and gender were included as covariates and removed during the analysis. Differences in the RBADI between individuals with specific diseases and the HC cohort were considered statistically significant at a *p*-value threshold of <0.05 after the FDR correction. The multivariate general linear models were implemented in IBM SPSS Statistics 27.

### 2.7. Classification Experiment for Stage Recognition of Neurodegenerative Diseases

To further validate the discriminative ability of the RBADI in early disease screening, we conducted a series of supervised experiments. Three-class classification tasks were employed: HCs vs. MCI vs. AD and HCs vs. pPD vs. PD. A diverse range of machine learning architectures was evaluated, encompassing traditional methods such as SVM, XGBoost, and Lasso regression. To ensure robust model evaluation, the subjects in both experimental groups were divided into a training set (80%) and a test set (20%). To address potential class imbalances within the training data, data augmentation techniques were employed. The performance of each classification task was rigorously assessed using a combination of metrics, including classification accuracy (ACC), sensitivity (SEN), specificity (SPE), and area under the curve (AUC). Three distinct input groups—the RBADI, brain age vectors (BAV), and BAG—were systematically established to compare the discriminative capacity of different metrics for identifying neurodegenerative disorders. Specifically, BAV represents a measure of brain aging with enhanced neurodegenerative disorder specificity [25], which was characterized by Shapley values in this study. BAG, calculated as the discrepancy between biological brain age and CA, serves as the most widely adopted evaluation metric for brain aging assessment. Age and gender information for each subject were included as additional input features.

The training and evaluation of all models were conducted on the Python (3.10.13) platform. SVM and Lasso regression were implemented using the scikit-learn (1.3.2) toolkit, and XGBoost was implemented using the xgboost (2.1.0) toolkit.

## 3. Results

### 3.1. Performance and Prediction of the Brain Age Model

**Figure 3:**
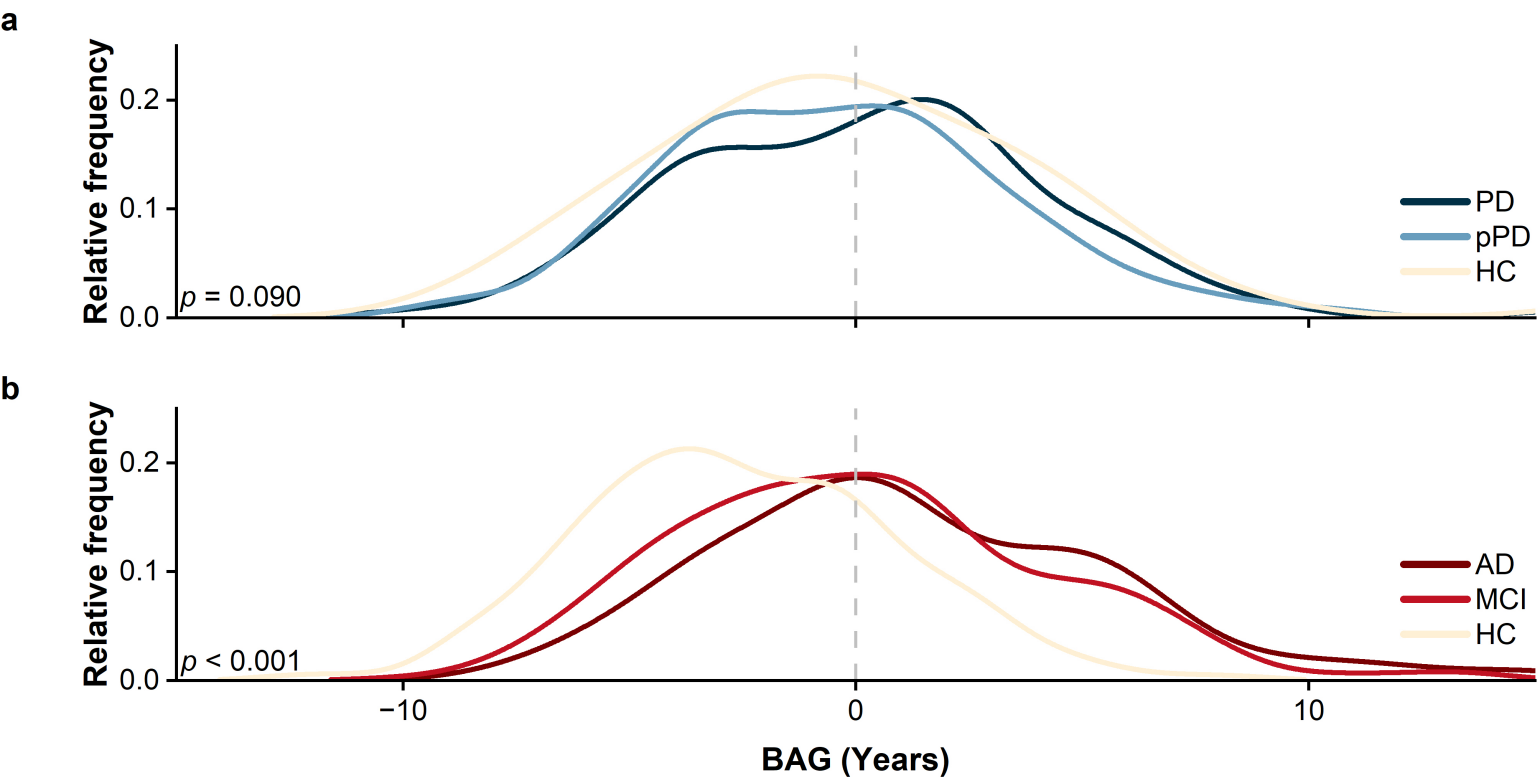
Differences in BAG across groups using a univariate linear regression method to examine intergroup BAG differences with age and sex removed as covariates. Part (**a**) shows the BAG distribution for the HC vs. pPD vs. PD groups, with the intergroup difference *p*-value reported as 0.090, indicating no significant differences between the groups. Part (**b**) represents the BAG distribution for the HC vs. MCI vs. AD groups, with the intergroup difference *p*-value reported as less than 0.001, indicating significant differences between the groups.

The advanced predictive performance of the 3D ds-FCRN has been demonstrated in previous studies. Table 2 presents the predictive performance of the model on different test datasets, while Figure 3 illustrates the distribution of BAGs for various specific diseases.

In the HCs vs. pPD vs. PD groups, there were no significant differences in BAG among the three groups (p=0.090), and the mean BAG values for the three groups were 0.08 (HCs), −0.44 (pPD), and 0.24 (PD); however, a marginally significant difference in BAG was observed between the PD and pPD groups (p=0.027). This may be attributed to the relatively small number of HCs and the imbalance in sample sizes across groups. In contrast, significant differences in BAG were observed among the HC vs. MCI vs. AD groups (p<0.001), with mean BAG values of −2.58 (HCs), 0.15 (MCI), and 1.27 (AD), respectively. This indicates that both AD and PD exhibit abnormal aging at the whole-brain level; however, the specific abnormal regions associated with each disease cannot be determined.

### 3.2. Regional-Level Contributions

**Figure 4:**
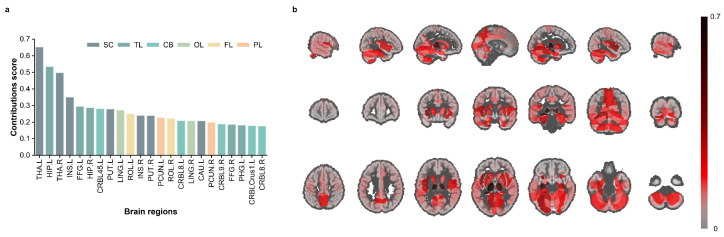
Regional-level contributions. Part (**a**) shows the specific contribution values for the top 20% brain regions; part (**b**) displays the contribution heatmap for all brain regions.

Benefiting from an efficient multi-stage Shapley values workflow, we calculated regional-level contributions in the strictly healthy subject group of the UKB test set. This analysis characterizes the contributions of different brain regions to the decision-making process of the brain age prediction model. Figure 4b presents a heatmap of contributions across various brain regions.

The brain regions in the SC and TL areas demonstrated important contributions to the decision-making process of the brain age prediction model, with the THA, HIP, and INS exhibiting particularly notable contributions.

### 3.3. Regional Brain Aging Patterns

**Figure 5:**
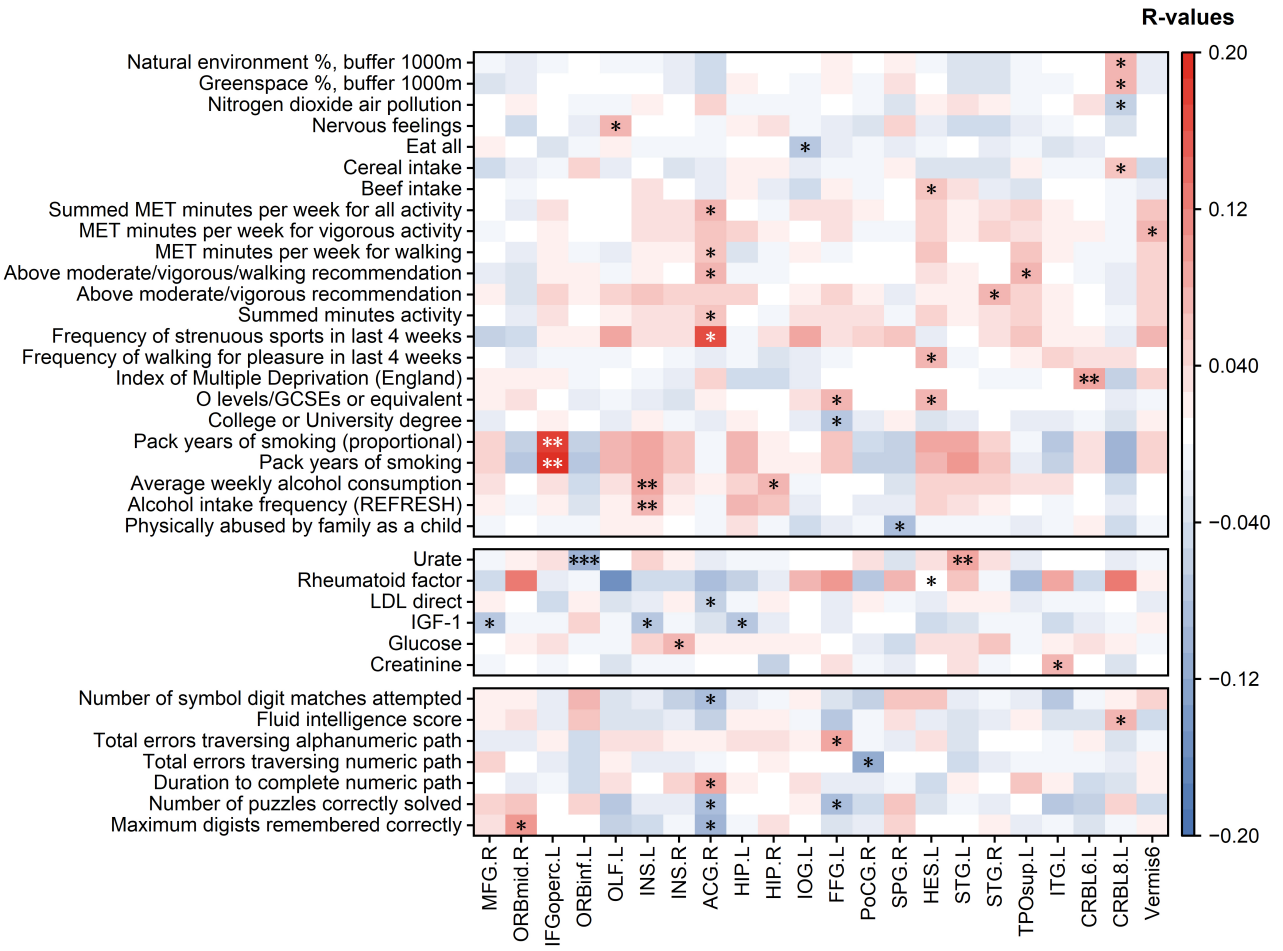
Heatmaps of the correlation analysis between brain region aging and lifestyle, blood biochemical markers, and cognitive abilities. Only the correlations marked with asterisk (*) are statistically significant, where * indicates p<0.05, ** indicates p<0.01, and *** indicates p<0.001.

Figure 5 illustrates the correlations between brain region aging and lifestyle, blood biochemical markers, and cognitive abilities, with only the correlations marked with an asterisk (*) being statistically significant. The statistical results, including *p*-values, R-values, and FDR-corrected *p*-values, are presented in Supplementary Tables S5, S6 and S7, respectively.

**Figure 6:**
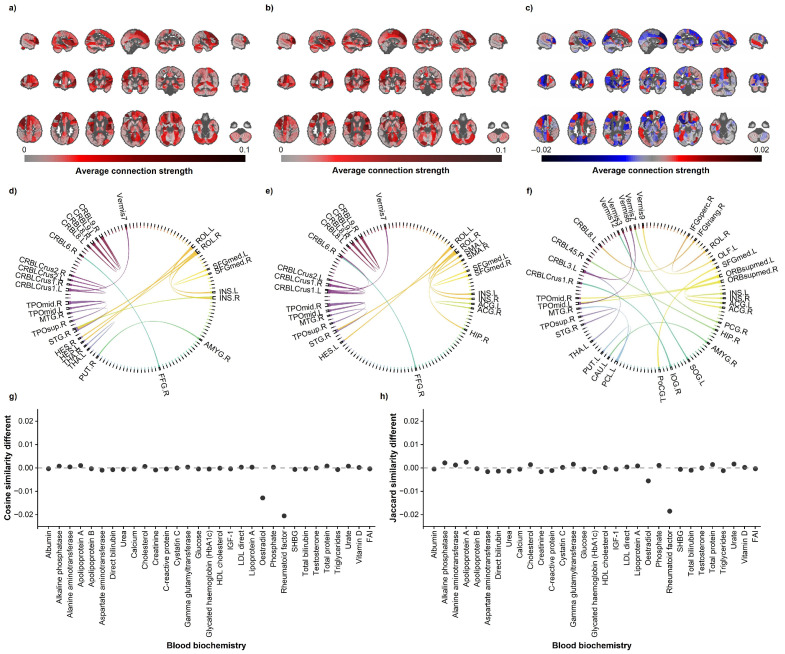
Aging networks. Part (**a**) denotes the average connection strength of the male aging network; part (**b**) represents the average connection strength of the female aging network; part (**c**) indicates the sex difference in aging network connectivity, calculated as the average connection strength of the male aging network minus that of the female aging network; part (**d**) shows the top 20 strongest connections in the male aging network; part (**e**) displays the top 20 strongest connections in the female aging network; part (**f**) illustrates the 20 connections with the most significant sex differences in aging networks; part (**g**) demonstrates the influence of blood biomarkers on sex-specific aging network similarity under cosine similarity metrics; part (**h**) reveals the impact of blood biomarkers on sex-specific aging network similarity measured by Jaccard similarity indices.

Figure 6a–c displays the mean connection strength of the male aging network, female aging network, and their differences, respectively. Both males and females exhibited higher mean connection strengths in most frontal and prefrontal brain regions, including ROL.R, IFGtriang.R, ROL.L, ORBmid.L, SFGmed.L, MFG.L, and ORBsup.R. Notably, the mean connection strength of INS in the female aging network was greater than that in males, while other brain regions showed comparable connection strengths between sexes. Figure 6d–f illustrate the top 20 strongest connections in the male aging network, female aging network, and the 20 most divergent connections between sexes, respectively. The male aging network demonstrated a higher proportion of cross-anatomical-region connections, whereas the female aging network exhibited stronger intra-anatomical-region connectivity. Figure 6g,h presents the effects of different blood biomarkers on sex-specific similarities in aging networks. Both similarity metrics revealed reduced male–female network similarity after controlling for estradiol or Rheumatoid factors as covariates.

**Figure 7:**
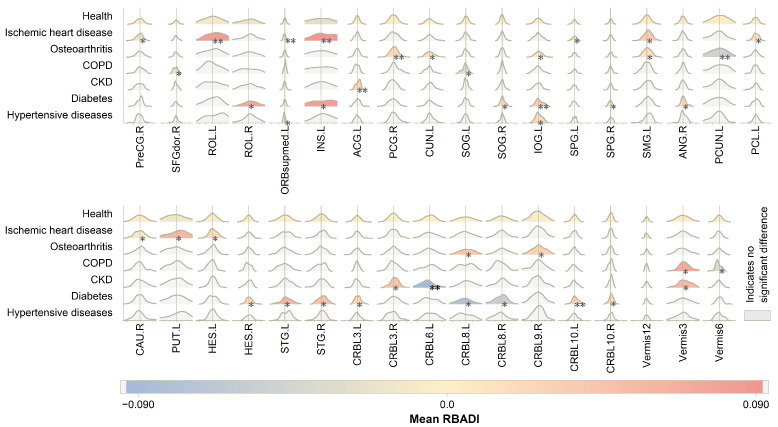
RBADI in chronic disease. * indicates p<0.05 and ** indicates p<0.01. Since no significant differences were found between the RBADI of individuals with chronic diseases and HCs after applying FDR correction, the results reported here are those without FDR correction.

Figure 7 demonstrates the impact of specific diseases on the RBADI, with different chronic diseases exhibiting significantly distinct aging patterns.

### 3.4. Classification Experiment for Stage Recognition of Neurodegenerative Diseases

Table 3 presents the performance of different machine learning models with varied inputs across various tasks, where the RBADI demonstrates the most superior overall performance in both AD and PD recognition. The ROC curves are displayed in Supplementary Figure S1.

## 4. Discussion

In this study, we introduced a more fine-grained RBADI for a post hoc interpretation of models and an analysis of lifestyle factors and disease progression. This metric was enabled by an efficient multi-stage Shapley values computational workflow that significantly reduces computational complexity, thereby facilitating sufficiently fine-grained exploration on deep learning models. The RBADI analysis utilized data from three cohorts: 2684 strictly healthy subjects and 592 chronic disease subjects from the UKB; 582 HCs, 432 MCI, and 252 AD patients from the ADNI; and 36 HC, 426 pPD, and 264 PD patients from the PPMI. Through rigorous and meticulous analysis, our investigation yielded the following key findings:

(1) The SHAP method is not the sole interpretability approach applied in brain age prediction models. In fact, a diverse array of interpretability techniques has been successfully implemented in these frameworks. In addition to conventional methods such as CAM and saliency map techniques, other advanced approaches including Layer-wise Relevance Propagation (LRP) algorithms [32,33], occlusion sensitivity analysis [34,35], and attention-score-based approaches [36,37] have been systematically employed and incorporated into model interpretation architectures. However, the strength of the SHAP method lies in its role as a model-agnostic post hoc explanatory approach. This means SHAP demonstrates strong versatility in interpretation, applicable regardless of the model architecture or data type employed.
(2) The multi-stage Shapley values computation workflow offers an effective approach for calculating Shapley values, as it constitutes a model-agnostic post hoc explanation method that enables modular application to any deep learning model. Compared with the original Shapley values algorithm’s $2^{n}$ complexity, this algorithm significantly reduces computational complexity, enabling the computation of Shapley values at the brain regional level. Taking the AAL116 template as an example, the original algorithm would require $2^{116}$ (equal to $8.3\times 10^{34}$) operations to obtain Shapley values for each brain region, making such computation completely infeasible for relatively complex deep learning models. Through our proposed approximation algorithm, the computational complexity is reduced to three sequential stages: $2^{8}$ (equal to $2.6\times 10^{2}$) operations in the first stage, $7\times 2^{2}$ (equal to $2.8\times 10^{1}$) operations in the second stage, and $2\times \left(2^{10}+2^{4}+2^{12}+2^{8}+2^{6}+2^{5}+2^{9}\right)+2^{8}$ (equal to $1.2\times 10^{4}$) operations in the third stage. This achieves a remarkable reduction of approximately $10^{30}$ orders of magnitude in computational complexity compared to the original algorithm. Taking the 3D ds-FCRN architecture employed in this study as an example, performing a single age prediction for 3276 subjects in the test set requires approximately 15 s during the post hoc explanation process. The approximate computational workflow necessitates $1.2\times 10^{4}$ operations with a total execution time of 52 h, while the standard Shapley value computation method would require $8.3\times 10^{34}$ operations, amounting to $2\times 10^{34}$ h of computational time. This efficient approximation algorithm has enabled the application of Shapley value interpretation methods in deep learning, thereby facilitating the construction of the RBADI. The framework achieves region-level aging interpretation and reveals significant correlations between anomalous aging and factors including lifestyle patterns, mental health status, cognitive performance, and chronic disease profiles.
(3) The prominent contributions of the THA and HIP in brain age prediction models highlight the central role of these regions in cerebral aging processes, identifying them as a key predictor of brain age in HCs. Notably, among all brain regions, THA demonstrates the highest contribution value and with left-lateralized characteristics. These findings align with previous research. For instance, Wang et al. reported that THA exhibits high predictive weights in structural MRI models, where reduced GM volume and increased WM volume serve as critical features for brain age prediction [38]. Similarly, Zhang et al. identified THA and the CAU as pivotal regions for brain age prediction across the adult lifespan through an occlusion analysis of brain network atlases, with the left hemisphere (particularly THA.L) showing more pronounced contributions [39]. A plausible explanation for this lateralization involves THA’s asymmetric structural development and aging patterns [40], coupled with the heightened sensitivity of THA.L-mediated language information processing to age-related changes [41,42]. Furthermore, HIP emerges as an exceptionally critical region within the TL. Responsible for memory formation and spatial navigation, HIP plays a vital role in cognitive processes vulnerable to aging [43]. This study further reveals the significant contribution of HIP, especially HIP.L, in brain age prediction frameworks.
(4) The RBADI model provides an analytical framework with higher spatial resolution for deciphering the intricate relationships between lifestyle factors, mental health, cognitive abilities, and accelerated brain aging. Our findings reveal a significant association between abnormal aging in the IFGoperc.L and smoking duration. Previous studies have demonstrated that smoking behavior affects pain regulation, sleep quality, and depressive/anxiety symptoms through inferior frontal gyrus-mediated neural mechanisms [44,45]. Specifically, Addicott et al. found that smokers exhibited significantly enhanced task-based functional connectivity between the left inferior frontal gyrus (including the opercular part) and auditory cortex during cognitive-affective distress tasks, suggesting that smokers may require stronger IFGoperc activation to cope with distress-related stimuli [44]. Furthermore, Chen et al. demonstrated that enhanced functional connectivity between the hypothalamus and right inferior frontal gyrus in smokers was associated with poorer PSQI sleep quality scores, potentially reflecting neural compensatory mechanisms to maintain alertness and attentional control [45]. Additionally, the strong correlation between INS and alcohol consumption may stem from alcohol-induced alterations in insular functional connectivity patterns that impair cognitive control and reinforce positive alcohol expectancy [46]. Notably, the insula also plays a pivotal regulatory role in the stress–alcohol use pathway [47]. In conclusion, compared with BAG, the RBADI demonstrates superior regional specificity in brain aging assessments.
(5) When employing BAG for identifying prodromal neurodegenerative disorders (AD and PD), although significant differences were observed in the HC vs. MCI vs. AD groups, its discriminative capability remained unsatisfactory in distinguishing the HC vs. pPD vs. PD cohorts. The classification model trained with BAG, gender, and age as input features demonstrated a poor performance, only marginally better than random guessing. Compared with the previously well-performing BAV, our proposed RBADI provides age-correlated metrics that not only exhibit stronger competitiveness in neurodegenerative disease screening but also substantiate the pivotal role of brain aging in the progression of neurodegenerative disorders.

However, this study is subject to several limitations:

(1) The dataset employed in this study is characterized by racial representativeness imbalance [48,49]. Although this phenomenon is prevalent across research domains, it may still compromise the representativeness of the RBADI analytical outcomes. Future investigations should prioritize the incorporation of datasets encompassing diverse ethnic populations to validate the generalizability of the RBADI. Additionally, consideration should be given to implementing multi-center data integration strategies to address potential representativeness limitations [50,51,52].
(2) The current study solely utilized single-modality T1 MRI data, which is inherently limited to providing structural information. The integration of multimodal imaging data not only further enhances the predictive performance of models but also offers additional perspectives for post hoc model interpretability. Therefore, the primary focus of subsequent research will be to incorporate multimodal data to enable a more comprehensive investigation.
(3) Although the rigorous data preprocessing protocols and stringent quality control criteria excluded low-quality T1 MRI data, this approach may limit the model’s generalizability to noise-corrupted data. Considering that adversarial examples containing noise can easily interfere with deep neural networks and compromise their performance [53], future developments in model design and interpretation modules should incorporate adversarial noise considerations to enhance the robustness of both the predictive models and explanatory methodologies.

## 5. Conclusions

This study introduces an innovative multi-stage Shapley value computational workflow that significantly reduces the computational complexity of traditional Shapley value algorithms, enabling the precise quantification of feature contributions at the brain region level even in complex deep learning models. Building upon this efficient computational framework, we developed the RBADI correlated with chronological age. The brain-region-level Shapley values elucidate the decision-making process of brain age prediction models, with the UK Biobank cohort analysis revealing the pivotal roles of the THA and HIP in brain aging processes.

With its sufficient granular analytical capability, the RBADI uncovers significant correlations between lifestyle factors, blood biomarkers, cognitive performance, and region-specific brain aging patterns. Notably, it identifies strong associations between IFGoperc and smoking duration, as well as between INS and alcohol consumption levels. Furthermore, the RBADI demonstrates an excellent performance in classifying prodromal neurodegenerative diseases, suggesting its potential as a novel biomarker for the early detection of neurodegenerative disorders. This systematic approach to interpreting model decisions through cooperative game theory provides new perspectives for both aging mechanism exploration and clinical applications.

## Figures and Tables

**Table 1:** Demographic information.

Data Set	UKB	ADNI			PPMI		
Category	HCs	HCs	MCI	AD	HCs	pPD	PD
Number of subjects	16,377	582	432	252	36	426	264
Female/male	8566/7811	346/236	188/244	125/127	16/20	219/217	109/155
Age (years)	63.74±7.34/ 64.85±7.66	70.56±6.45/ 72.31±5.85	71.22±7.05/ 72.99±5.77	72.30±6.45/ 73.12±6.77	63.44±7.89/ 64.10±8.71	63.54±6.37/ 65.69±7.05	63.71±8.82/ 65.08±8.06

**Table 2:** Model’s performance on different test datasets.

Data Set	UKB	ADNI			PPMI		
Category	HCs	HCs	MCI	AD	HCs	pPD	PD
MAE	2.43±1.88	3.56±2.52	3.32±2.51	3.52±2.97	3.51±3.20	3.06±2.24	3.28±2.54
$R^{2}$	0.83	0.52	0.58	0.52	0.68	0.69	0.76

**Table 3:** Classification performance of different machine learning models across various tasks. Optimal performance under different input metrics is highlighted in bold.

	Task	HCs vs. MCI vs. AD			HCs vs. pPD vs. PD		
Model	Performance\Input	RBADI	BAV	BAG	RBADI	BAV	BAG
SVM	ACC	0.7969±0.0215	0.7890±0.0101	0.5323±0.0195	0.8945±0.0127	0.8890±0.0091	0.3192±0.0672
	SEN	0.7462±0.0373	0.7283±0.0083	0.5297±0.0292	0.6866±0.0468	0.6660±0.0505	0.2799±0.0640
	SPE	0.9085±0.0110	0.9039±0.0038	0.7670±0.0106	0.9448±0.0065	0.9416±0.0045	0.6668±0.0156
	AUC	0.9166±0.0093	0.9123±0.0044	0.7256±0.0191	0.8341±0.0251	0.8469±0.0356	0.4544±0.0355
XGboost	ACC	0.8047±0.0163	0.8031±0.0119	0.4913±0.0204	0.9479±0.0055	0.9479±0.0055	0.5151±0.0361
	SEN	0.7255±0.0206	0.7194±0.0146	0.4584±0.0163	0.6643±0.0031	0.6643±0.0031	0.3479±0.0291
	SPE	0.9082±0.0073	0.9069±0.0052	0.7363±0.0103	0.9597±0.0019	0.9594±0.0023	0.6940±0.0233
	AUC	0.9173±0.0076	0.9174±0.0066	0.6699±0.0288	0.8470±0.0302	0.9052±0.0278	0.4785±0.0404
Lasso	ACC	0.8008±0.0170	0.8000±0.0125	0.5323±0.0204	0.8795±0.0206	0.8767±0.0229	0.3329±0.0210
	SEN	0.7489±0.0339	0.7419±0.0173	0.5279±0.0303	0.6518±0.0338	0.6585±0.0522	0.2935±0.0436
	SPE	0.9100±0.0092	0.9089±0.0058	0.7662±0.0101	0.9376±0.0070	0.9373±0.0102	0.6732±0.0163
	AUC	0.9204±0.0083	0.9149±0.0034	0.7235±0.0200	0.8606±0.0129	0.8734±0.0271	0.4701±0.0314

## Data Availability

The datasets analyzed in this study were obtained from three publicly accessible repositories: (1) UK Biobank (UKB) (http://www.ukbiobank.ac.uk/register-apply/ (accessed on 10 August 2023)) through approved application procedures; (2) the Alzheimer’s Disease Neuroimaging Initiative (ADNI) (https://adni.loni.usc.edu/data-samples/adni-data/#AccessData (accessed on 15 April 2024)) following standard data request protocols; and (3) the Parkinson’s Progression Markers Initiative (PPMI) (https://www.ppmi-info.org/access-data-specimens/download-data (accessed on 15 April 2024)) via their designated access platform. The processed datasets and analytical outputs generated during this research are available from the corresponding author upon reasonable request, subject to compliance with institutional data sharing agreements and ethical review requirements.

## Supplementary Materials

The following supporting information can be downloaded at https://www.mdpi.com/article/10.3390/bioengineering12060607/s1: Supplementary Figure S1: ROC curves of classification results (class 0: HCs; class 1: prodromal neurodegenerative group; class 2: neurodegenerative group); Supplementary Table S1: ICD-10 codes used to exclude subjects with psychiatric disorders; Supplementary Table S2: The division of brain regions across different stages; Supplementray Table S3: Abbreviations for brain regions; Supplementary Table S4: ICD10 codes used for chronic disease subject screening; Supplementary Table S5: UKB Field IDs and correlation analysis results for the 146 lifestyle factors; Supplementary Table S6: UKB Field IDs and correlation analysis results for the 31 blood biochemical indicators; Supplementary Table S7: UKB Field IDs and correlation analysis results for the 32 cognitive ability indicators.

## References

[1] Franke K., Gaser C. (2019). Ten years of BrainAGE as a neuroimaging biomarker of brain aging: What insights have we gained?. Front. Neurol..

[2] Cole J.H., Poudel R.P., Tsagkrasoulis D., Caan M.W., Steves C., Spector T.D., Montana G. (2017). Predicting brain age with deep learning from raw imaging data results in a reliable and heritable biomarker. NeuroImage.

[3] Franke K., Ziegler G., Klöppel S., Gaser C., Alzheimer’s Disease Neuroimaging Initiative (2010). Estimating the age of healthy subjects from T1-weighted MRI scans using kernel methods: Exploring the influence of various parameters. NeuroImage.

[4] Lemaitre H., Goldman A.L., Sambataro F., Verchinski B.A., Meyer-Lindenberg A., Weinberger D.R., Mattay V.S. (2012). Normal age-related brain morphometric changes: Nonuniformity across cortical thickness, surface area and gray matter volume?. Neurobiol. Aging.

[5] Long X., Liao W., Jiang C., Liang D., Qiu B., Zhang L. (2012). Healthy aging: An automatic analysis of global and regional morphological alterations of human brain. Acad. Radiol..

[6] Coupé P., Manjón J.V., Mansencal B., Tourdias T., Catheline G., Planche V. (2022). Hippocampal-amygdalo-ventricular atrophy score: Alzheimer disease detection using normative and pathological lifespan models. Hum. Brain Mapp..

[7] Coupé P., Manjón J.V., Lanuza E., Catheline G. (2019). Lifespan changes of the human brain in Alzheimer’s disease. Sci. Rep..

[8] Cole J.H., Franke K. (2017). Predicting age using neuroimaging: Innovative brain ageing biomarkers. Trends Neurosci..

[9] Franke K., Gaser C. (2012). Longitudinal changes in individual BrainAGE in healthy aging, mild cognitive impairment, and Alzheimer’s disease. GeroPsych.

[10] Gaser C., Franke K., Klöppel S., Koutsouleris N., Sauer H., Alzheimer’s Disease Neuroimaging Initiative (2013). BrainAGE in mild cognitive impaired patients: Predicting the conversion to Alzheimer’s disease. PLoS ONE.

[11] Beheshti I., Mishra S., Sone D., Khanna P., Matsuda H. (2019). T1-weighted MRI-driven brain age estimation in Alzheimer’s disease and Parkinson’s disease. Aging Dis..

[12] Johnson K.A., Fox N.C., Sperling R.A., Klunk W.E. (2012). Brain imaging in Alzheimer disease. Cold Spring Harb. Perspect. Med..

[13] Lewis M.M., Du G., Lee E.Y., Nasralah Z., Sterling N.W., Zhang L., Wagner D., Kong L., Tröster A.I., Styner M. (2016). The pattern of gray matter atrophy in Parkinson’s disease differs in cortical and subcortical regions. J. Neurol..

[14] Nguyen H.D., Clément M., Mansencal B., Coupé P. (2024). Brain structure ages—A new biomarker for multi-disease classification. Hum. Brain Mapp..

[15] Kaufmann T., van der Meer D., Doan N.T., Schwarz E., Lund M.J., Agartz I., Alnæs D., Barch D.M., Baur-Streubel R., Bertolino A. (2019). Common brain disorders are associated with heritable patterns of apparent aging of the brain. Nat. Neurosci..

[16] Bloch L., Friedrich C.M. Developing a Machine Learning Workflow to Explain Black-box Models for Alzheimer’s Disease Classification. Proceedings of the 14th International Joint Conference on Biomedical Engineering Systems and Technologies, SCITEPRESS—Science and Technology Publications.

[17] Li X., Zhou Y., Dvornek N.C., Gu Y., Ventola P., Duncan J.S. Efficient Shapley explanation for features importance estimation under uncertainty. Proceedings of the Medical Image Computing and Computer Assisted Intervention—MICCAI 2020: 23rd International Conference.

[18] Besson P., Parrish T., Katsaggelos A.K., Bandt S.K. (2021). Geometric deep learning on brain shape predicts sex and age. Comput. Med. Imaging Graph..

[19] Khosla M., Jamison K., Kuceyeski A., Sabuncu M.R. (2019). Ensemble learning with 3D convolutional neural networks for functional connectome-based prediction. NeuroImage.

[20] Hong J., Yun H.J., Park G., Kim S., Ou Y., Vasung L., Rollins C.K., Ortinau C.M., Takeoka E., Akiyama S. (2021). Optimal method for fetal brain age prediction using multiplanar slices from structural magnetic resonance imaging. Front. Neurosci..

[21] Zhou B., Khosla A., Lapedriza A., Oliva A., Torralba A. Learning deep features for discriminative localization. Proceedings of the IEEE Conference on Computer Vision and Pattern Recognition.

[22] Simonyan K., Vedaldi A., Zisserman A. (2013). Deep inside convolutional networks: Visualising image classification models and saliency maps. arXiv.

[23] Salih A., Galazzo I.B., Raisi-Estabragh Z., Petersen S.E., Gkontra P., Lekadir K., Menegaz G., Radeva P. A new scheme for the assessment of the robustness of explainable methods applied to brain age estimation. Proceedings of the 2021 IEEE 34th International Symposium on Computer-Based Medical Systems (CBMS).

[24] Ballester P.L., Suh J.S., Ho N.C., Liang L., Hassel S., Strother S.C., Arnott S.R., Minuzzi L., Sassi R.B., Lam R.W. (2023). Gray matter volume drives the brain age gap in schizophrenia: A SHAP study. Schizophrenia.

[25] Ran C., Yang Y., Ye C., Lv H., Ma T. (2022). Brain age vector: A measure of brain aging with enhanced neurodegenerative disorder specificity. Hum. Brain Mapp..

[26] Wu Y., Gao H., Zhang C., Ma X., Zhu X., Wu S., Lin L. (2024). Machine learning and deep learning approaches in lifespan brain age prediction: A comprehensive review. Tomography.

[27] Sajedi H., Pardakhti N. (2019). Age prediction based on brain MRI image: A survey. J. Med. Syst..

[28] Wang J., Knol M.J., Tiulpin A., Dubost F., de Bruijne M., Vernooij M.W., Adams H.H., Ikram M.A., Niessen W.J., Roshchupkin G.V. (2019). Gray matter age prediction as a biomarker for risk of dementia. Proc. Natl. Acad. Sci. USA.

[29] Dufumier B., Gori P., Battaglia I., Victor J., Grigis A., Duchesnay E. (2021). Benchmarking cnn on 3d anatomical brain mri: Architectures, data augmentation and deep ensemble learning. arXiv.

[30] Wu Y., Zhang C., Ma X., Zhu X., Lin L., Tian M. (2025). ds-FCRN: Three-dimensional dual-stream fully convolutional residual networks and transformer-based global–local feature learning for brain age prediction. Brain Struct. Funct..

[31] Wang L., Li J. (2021). The value of serum-free androgen index in the diagnosis of polycystic ovary syndrome: A systematic review and meta-analysis. J. Obstet. Gynaecol. Res..

[32] Chen J.V., Chaudhari G., Hess C.P., Glenn O.A., Sugrue L.P., Rauschecker A.M., Li Y. (2022). Deep Learning to Predict Neonatal and Infant Brain Age from Myelination on Brain MRI Scans. Radiology.

[33] Hofmann S.M., Beyer F., Lapuschkin S., Goltermann O., Loeffler M., Müller K.R., Villringer A., Samek W., Witte A.V. (2022). Towards the interpretability of deep learning models for multi-modal neuroimaging: Finding structural changes of the ageing brain. NeuroImage.

[34] Jiang H., Lu N., Chen K., Yao L., Li K., Zhang J., Guo X. (2020). Predicting brain age of healthy adults based on structural MRI parcellation using convolutional neural networks. Front. Neurol..

[35] Kuchcinski G., Rumetshofer T., Zervides K.A., Lopes R., Gautherot M., Pruvo J.P., Bengtsson A.A., Hansson O., Jönsen A., Sundgren P.C.M. (2023). MRI BrainAGE demonstrates increased brain aging in systemic lupus erythematosus patients. Front. Aging Neurosci..

[36] Shi W., Yan G., Li Y., Li H., Liu T., Sun C., Wang G., Zhang Y., Zou Y., Wu D. (2020). Fetal brain age estimation and anomaly detection using attention-based deep ensembles with uncertainty. NeuroImage.

[37] Cai H., Gao Y., Liu M. (2022). Graph transformer geometric learning of brain networks using multimodal MR images for brain age estimation. IEEE Trans. Med. Imaging.

[38] Wang Q., Hu K., Wang M., Zhao Y., Liu Y., Fan L., Liu B. (2021). Predicting brain age during typical and atypical development based on structural and functional neuroimaging. Hum. Brain Mapp..

[39] Zhang X., Pan Y., Wu T., Zhao W., Zhang H., Ding J., Ji Q., Jia X., Li X., Lee Z. (2024). Brain age prediction using interpretable multi-feature-based convolutional neural network in mild traumatic brain injury. NeuroImage.

[40] Ahsan R.L., Allom R., Gousias I.S., Habib H., Turkheimer F.E., Free S., Lemieux L., Myers R., Duncan J.S., Brooks D.J. (2007). Volumes, spatial extents and a probabilistic atlas of the human basal ganglia and thalamus. NeuroImage.

[41] Llano D.A. (2013). Functional imaging of the thalamus in language. Brain Lang..

[42] Federmeier K.D., Kutas M. (2005). Aging in context: Age-related changes in context use during language comprehension. Psychophysiology.

[43] Bird C.M., Burgess N. (2008). The hippocampus and memory: Insights from spatial processing. Nat. Rev. Neurosci..

[44] Addicott M.A., Oliveto A.H., Daughters S.B. (2023). Smoking status affects cognitive, emotional and neural-connectivity response to distress-inducing auditory feedback. Drug Alcohol Depend..

[45] Chen Y., Chaudhary S., Li G., Fucito L.M., Bi J., Li C.S.R. (2024). Deficient sleep, altered hypothalamic functional connectivity, depression and anxiety in cigarette smokers. NeuroImage Rep..

[46] Le T.M., Malone T., Li C.S.R. (2022). Positive alcohol expectancy and resting-state functional connectivity of the insula in problem drinking. Drug Alcohol Depend..

[47] Bach P., Zaiser J., Zimmermann S., Gessner T., Hoffmann S., Gerhardt S., Berhe O., Bekier N.K., Abel M., Radler P. (2024). Stress-Induced Sensitization of Insula Activation Predicts Alcohol Craving and Alcohol Use in Alcohol Use Disorder. Biol. Psychiatry.

[48] Sudlow C., Gallacher J., Allen N., Beral V., Burton P., Danesh J., Downey P., Elliott P., Green J., Landray M. (2015). UK biobank: An open access resource for identifying the causes of a wide range of complex diseases of middle and old age. PLoS Med..

[49] Petersen R.C., Aisen P.S., Beckett L.A., Donohue M.C., Gamst A.C., Harvey D.J., Jack C.R., Jagust W.J., Shaw L.M., Toga A.W. (2010). Alzheimer’s Disease Neuroimaging Initiative (ADNI). Neurology.

[50] Leonardsen E.H., Peng H., Kaufmann T., Agartz I., Andreassen O.A., Celius E.G., Espeseth T., Harbo H.F., Høgestøl E.A., De Lange A.M. (2022). Deep neural networks learn general and clinically relevant representations of the ageing brain. NeuroImage.

[51] Feng X., Lipton Z.C., Yang J., Small S.A., Provenzano F.A., Alzheimer’s Disease Neuroimaging Initiative, Australian Imaging Biomarkers and Lifestyle Flagship Study of Ageing, Frontotemporal Lobar Degeneration Neuroimaging Initiative (2020). Estimating brain age based on a uniform healthy population with deep learning and structural magnetic resonance imaging. Neurobiol. Aging.

[52] Levakov G., Rosenthal G., Shelef I., Raviv T.R., Avidan G. (2020). From a deep learning model back to the brain—Identifying regional predictors and their relation to aging. Hum. Brain Mapp..

[53] Kwon H. (2024). AudioGuard: Speech Recognition System Robust against Optimized Audio Adversarial Examples. Multimed. Tools Appl..

