# Supplementary material for: Regional Brain Aging Disparity Index: Region-Specific Brain Aging State Index for Neurodegenerative Diseases and Chronic Disease Specificity
Source: Bioengineering (Basel). 2025 Jun 3;12(6):607. doi: 10.3390/bioengineering12060607 (PMC12189761; doi:10.3390/bioengineering12060607)
Supplement: Supplementary file 1 [file bioengineering-12-00607-s001.zip › Supplementary Figure.pdf]

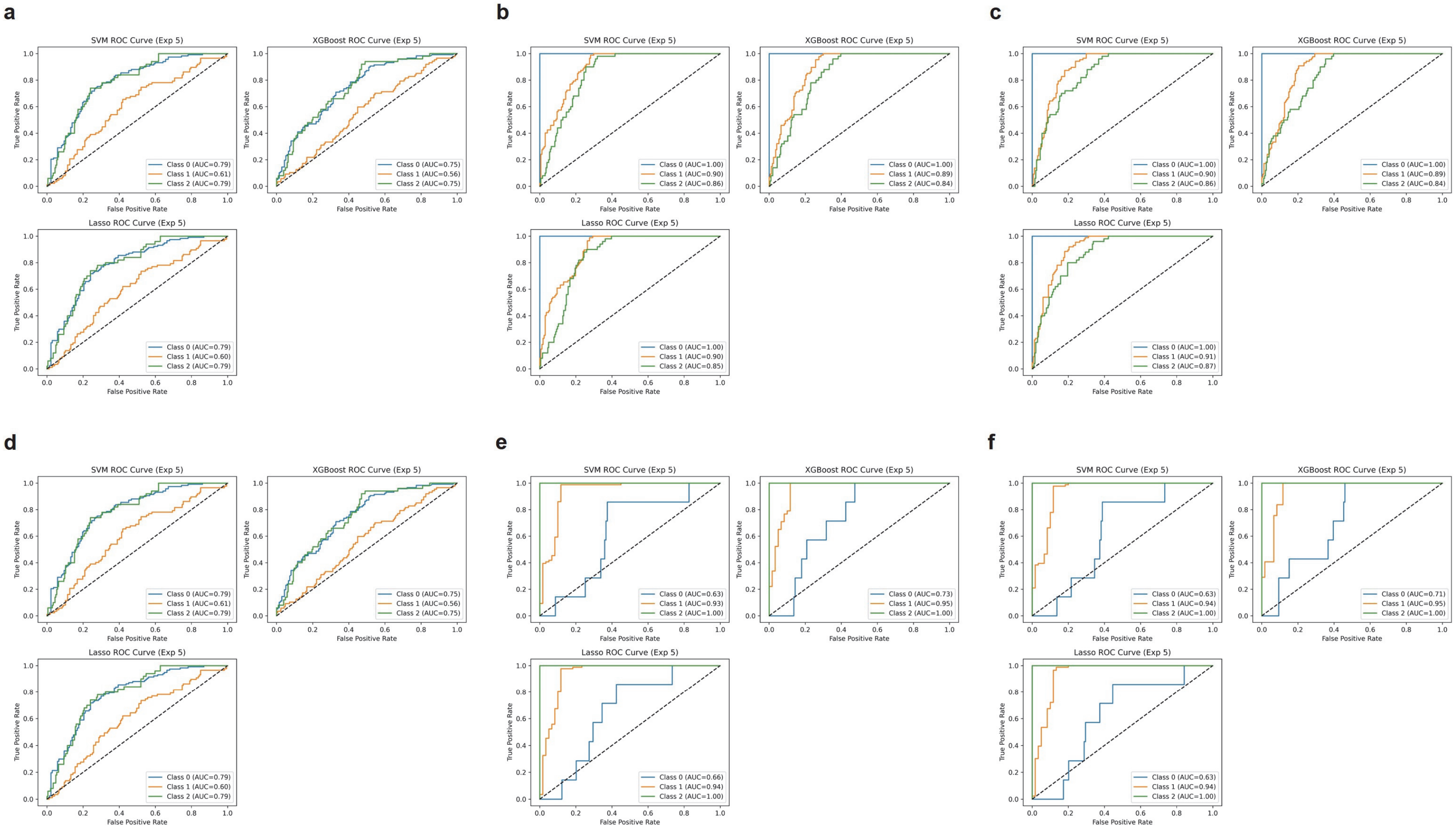

Supplementary Figure S1. ROC Curves of Classification Results (Class 0: HCs; Class 1: Prodromal Neurodegenerative Group; Class 2: Neurodegenerative Group). Parts a, b, c Represent ROC Curves for HCs vs. MCI vs. AD Classification Tasks Using BAG, BAV, and RBADI as Input Features, respectively; Parts d, e, f Represent ROC Curves for HCs vs. pPD vs. PD Classification Tasks Using BAG, BAV, and RBADI as Input Features, respectively.
